# Beyond Cleansing: Ecosystem Services Related to Phytoremediation

**DOI:** 10.3390/plants12051031

**Published:** 2023-02-24

**Authors:** Werther Guidi Nissim, Stefano Castiglione, Francesco Guarino, Maria Chiara Pastore, Massimo Labra

**Affiliations:** 1 Department of Biotechnology and Biosciences, University of Milano Bicocca, Piazza della Scienza 2, 20126 Milan, Italy; 2 Department of Chemistry and Biology “A. Zambelli”, University of Salerno, Via G. Paolo II n◦ 132, 84084 Fisciano, Italy; 3 Politecnico di Milano, Department of Architecture and Urban Studies, Via Bonardi 3, 20133 Milano, Italy; 4 National Biodiversity Future Center (NBFC), 90133 Palermo, Italy

**Keywords:** phytotechnologies, phytoremediation, ecosystem services, nature-based solution, green transition

## Abstract

Phytotechnologies used for cleaning up urban and suburban polluted soils (i.e., brownfields) have shown some weakness in the excessive extent of the timeframe required for them to be effectively operating. This bottleneck is due to technical constraints, mainly related to both the nature of the pollutant itself (e.g., low bio-availability, high recalcitrance, etc.) and the plant (e.g., low pollution tolerance, low pollutant uptake rates, etc.). Despite the great efforts made in the last few decades to overcome these limitations, the technology is in many cases barely competitive compared with conventional remediation techniques. Here, we propose a new outlook on phytoremediation, where the main goal of decontaminating should be re-evaluated, considering additional ecosystem services (ESs) related to the establishment of a new vegetation cover on the site. The aim of this review is to raise awareness and stress the knowledge gap on the importance of ES associated with this technique, which can make phytoremediation a valuable tool to boost an actual green transition process in planning urban green spaces, thereby offering improved resilience to global climate change and a higher quality of life in cities. This review highlights that the reclamation of urban brownfields through phytoremediation may provide several regulating (i.e., urban hydrology, heat mitigation, noise reduction, biodiversity, and CO_2_ sequestration), provisional (i.e., bioenergy and added-value chemicals), and cultural (i.e., aesthetic, social cohesion, and health) ESs. Although future research should specifically be addressed to better support these findings, acknowledging ES is crucial for an exhaustive evaluation of phytoremediation as a sustainable and resilient technology.

## 1. Introduction

The use of green living plants to address pollution is a growing option for environmental management of polluted sites, for it encompasses both environmental (solar-driven technology) and economical (cheaper than most conventional technologies) features, which are both required to meet sustainability and resilience goals for modern societies [1]. This phytotechnology shows high potential for many sites, especially in emerging countries, where it could represent a rather inexpensive reclamation technique compared to conventional, expensive, engineered technologies [2]. However, the technology shows some weaknesses that still hinder the application on a large scale. Although constraints are specific to each pollutant, low plant tolerance to environmental toxicants, long life span to be effective, and pollutant availability for the plant roots are the most common for different phytoremediation approaches [3]. Despite advances in the understanding of the mechanisms regulating the relationship between plants and pollutants and the subsequent new strategies to enhance the whole reclamation process, the improvements in the functionality of the technology at a large, field scale are slow [4]. However, the use of a green approach based on living plants should imply that phytoremediation will also provide additional environmental benefits, which must be assessed in the future. The current review aims to highlight the opportunities that the phytoremediation of urban brownfields offers in terms of ecosystem services (ESs). The goal of this review is to provide a critical outlook on the potential production of ESs during brownfield reclamation when a phyto-technological approach is used.

## 2. Lights and Shadows of Phytomanagement of Brownfields

### 2.1. The Dual Identity of Brownfields: Challenges and Opportunities

Over the last several decades, cities in many parts of the world have been subjected to a dramatic rise in population. Currently, about 55% of the world’s population lives in urban areas, and this proportion is expected to increase to 68% by 2050 [5]. This continuous increase in urbanization has induced many economic activities (i.e., industries) to shift from urban to suburban areas to leave space for new settlements. The displacement of industries from the city centre to peri urban areas often leaves the inner core typically with innumerable underutilized or vacant industrial sites. This has resulted in numerous sites that remain derelict or underused due to land-use restrictions based on concerns related to contamination by hazardous substances [6]. Although there is still a very active debate about how to define these sites [7], these areas are commonly referred to as brownfields [8].

Although brownfields are ubiquitous, their precise extent is not easy to quantify on a global scale, mainly due to disagreement about their definitions [9]. A survey carried out in 2001 in several EU countries highlighted that the extent of these lands varied greatly among countries (e.g., 128,000 ha in Germany; 39,000 in the United Kingdom; 20,000 in France; 13,000 in Italy; and 10,000 in the Netherland) [10]. In the USA, more than 500,000 brownfield sites still need to be redeveloped. Most brownfields are concentrated in an urban context. For instance, in the USA, 5–10% of the urban land is classified as brownfield, and the percentage in cities of the Northeast and Midwest (Rustbelt) states is even higher [11]. Brownfield contamination is generally located in both the soil and the groundwater and is due to either organic or inorganic toxicants, or in most cases, both [12]. Among inorganic contaminants, trace elements (i.e., Al, B, Ca, Cd, Co, Cr, Cu, Fe, K, Mg, Mn, Na, P, Pb, Si, Ti, V, and Zn) are very common, along with asbestos [13], whereas the main organic compounds are represented by polycyclic aromatic hydrocarbons (PAHs), polychlorinated biphenyls (PCBs), and petroleum hydrocarbons (PHCs) [14]. At present, the elevated level of pollution in urban brownfields can be attributed to several cooccurring factors, including (i) transportation, (ii) commercial and industrial emissions, and (iii) domestic activities [15]. 

In many countries, the redevelopment of urban brownfields is considered socially, economically, environmentally, and culturally important for city planning and a valuable tool to counteract urban sprawl [16]. However, brownfields oftentimes pose health risks for inhabitants. Some studies have reported brownfield exposure to be correlated with regional inequalities in mortality and morbidity in different regions within the UK [17]. Specifically, living close to brownfield sites may result in significantly lower naive T-cell production, suggesting accelerated immune system aging for people living near these sites [18]. Current strategies for brownfield reclamation include saturated zone and vadose zone technologies [19]. While the former approach is recommended when contamination affects both soil and groundwater, vadose technologies are more adapted for polluted groundwater. Pump-and-treat, reactive walls, air sparging, dual phase extraction, flushing, bioremediation, electrokinetic, and immobilization are the most common saturated zone technologies available on the market. Soil excavation, followed by either landfill disposal or treatment (i.e., soil washing, solvent extraction, electrokinetic, thermal desorption, incineration, vitrification, or bioremediation), is the most popular ex situ vadose approach [20]. Unfortunately, this very efficient approach shows many weaknesses related to its high operational costs and low environmental sustainability. From this perspective, on-site remediation techniques seem a more attractive solution. In situ vadose techniques include several highly engineered approaches, some of which (e.g., soil vapor extraction, soil flushing, electrokinetic, soil heating, vitrification, and solidification) often rely on non-renewable energy sources [21] with high environmental footprints [22]. For instance, it has been shown that 2.7 million tons of CO_2_ were produced using a “dig and haul” approach to remediate a single brownfield in New Jersey (USA), which is equal to 2% of the annual CO_2_ emissions for the entire state [23]. By contrast, phytoremediation approaches show high potential for their small environmental footprints, low operational costs, and high social acceptance [24]. 

### 2.2. From Bench to Field: Success and Challenges of Brownfield Phytoremediation

Since the beginning, phytoremediation received positive feedback, provided by the first studies showing its high potential for specific pollutants to be either taken up and accumulated or degraded by specific plants [25] (Table 1). However, like many other environmental technologies, the scaling up of phytoremediation from lab and mesocosm to actual field conditions has often resulted in different outputs, ranging from complete success to almost complete failure, thereby attenuating the initial enthusiasm. This variability in the performance response at field scale is due to co-occurring factors, including the type, status (e.g., bioavailability for trace elements) [26], and concentrations of toxicant(s) [27]; soil chemical and physical properties (pH, conductivity, texture, porosity, nutrient levels, and presence of soil microorganisms) [28]; plant species; rate of plant growth; and climatic conditions [29]. 

One of the most successful full-scale phytoremediation approaches in the field is a particular type of degradation technique known as phytometabolism. In this case, the environmental toxicants are also plant nutrients (e.g., inorganic elements, such as N and P), which, for this reason, are directly metabolized and incorporated into the plant’s biomass. This is the case of vegetation filter systems for the treatment of municipal wastewater. A recent review reported that in global terms, vegetation filters, which are mainly (70%) constituted by tree species belonging to the Salicaceae family, show average removal rates of about 78% for N and 80% for P [60], making this technology a suitable green tool especially suited for scattered populations or isolated buildings lacking connection to sewer systems. On the opposite side are the techniques based on the extraction of some inorganic pollutants that, in some cases, might take a considerable amount of time to be removed offsite.

The removal from soil of trace elements (both metals and metalloids) by plants is one clear example of phytotechnology that shows both high potential and interest and huge operational constraints. This approach has been extensively used at full field scale in different regions. The early field applications reported promising results for *Brassica juncea* combined with soil amendments on a lead-polluted brownfield [61] and for *Buchloe dactyloides* for naphthalene phytoremediation [62]. However, in this period, scientists still claimed that there was a substantial need for more demonstration projects to prove the efficiency of green technologies in the field from a long-term perspective [63]. These circumstances have led to the development of actual rehabilitation projects at field scale. Most initiatives have assessed the potential of several approaches for the cleansing of metal-contaminated brownfields, including phytoextraction by different species [64,65,66] and phytostabilization [67,68]. For example, the GREENLAND project funded by the European Commission (FP7) established a large-scale, field demonstration network, where new approaches and financial aspects related to phytoremediation were investigated [69].

Another field-scale project was developed in Rozelle (Australia), where *Brassica juncea* provided positive results, taking up significant amounts of lead from contaminated soil [70]. Promising results were also obtained in the “Opération Tournesol”, a Belgian-led rehabilitation project in Brussels, where *Noccaea caerulescens* showed positive results for cadmium and zinc phytoextraction [71]. Some woody species (i.e., willow and poplar) have also shown a notable capacity to remediate soil contaminated by trace elements on a former brownfield site in Detroit (USA), but additional time is required for validation [72].

Hence, despite intensive research over the past few years, trace element pollution continues to be challenging, and to date, there is still not a perfect phytotechnology for cleaning up and restoring soils within a reasonable timeframe. Different models have predicted that under ideal conditions, plants with an average dry biomass yield of 10 Mg ha^−1^ yr^−1^ should show bioaccumulation coefficients higher than 7.4 to reduce the total metal concentration by 50% in 25 years of phytoextraction [73], a condition which rarely occurs in nature, even for hyperaccumulator plants [74]. Time-consuming phytoextraction has been addressed by several strategies aimed at intensifying the efficiency of phytoremediation, thereby reducing the overall duration of the process. These include synergistic growth of plants and plant growth-promoting microorganisms [75], the use of chelating agents and soil amendments [76], co-planting different species [77], and the use of transgenic plants [26]. Despite these efforts, phytoextraction yields are expected to be rather low and the time for operating still too long to compete with conventional remediation techniques, making its application at field scale less attractive. In addition, during the recent decade, some concerns have been raised about the biomass issued from the process, which in some cases may contain significant amounts of toxicants, thereby representing a waste to be managed [78]. However, new promising strategies, where polluted biomass is used as feedstock or thermo- and biochemical compounds converted into biofuels, are now under assessment [79].

## 3. Ecosystem Services: The Known and Unknown Aspects Related to Phytoremediation of Brownfields

**Figure 1:**
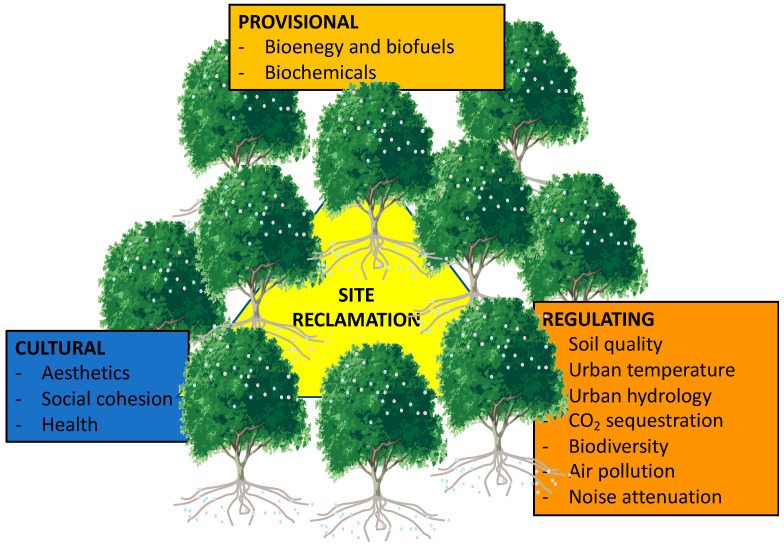
The main components of ecosystem services related to phytomanagement.

The most popular definition of ecosystem services (ESs) is a hybrid ecological–economic approach, directly linking an ecosystem’s functions and processes and the benefits derived for humans [80]. The most common ESs are regulating, provisioning, supporting, and cultural services. Regulating services are the benefits provided by ecosystems that moderate natural phenomena. These are the benefits obtained from the regulation of ecosystem processes, and they include flood protection, climate regulation, water purification, air quality maintenance, and biodiversity, all of which contribute to human well-being in cities [81]. Provisioning services are those related to the production of goods from any natural process. Cultural ESs are non-material benefits obtained from ecosystems (i.e., cultural diversity, spiritual and religious values, knowledge systems, educational values, inspiration, aesthetic values, social relations, sense of place, cultural heritage values, recreation, and ecotourism) that people may take advantage of [82]. The assessment of ESs in brownfield redevelopment has been assessed in many contexts, but most studies refer to green urban brownfields. These spaces are generated when an unsealed brownfield undergoes natural processes of ecological succession, thereby leading to a particular type of urban vegetation [83]. These brownfields are thought to have the potential to provide a wide range of ESs and that the differences in their extent depends on the specific structure and composition of the vegetation cover [84]. Similar conclusions have been drawn for soft brownfield re-use approaches, where the new established green ecosystems can provide multiple ESs to improve the urban environment, citizen health, and quality of life [85]. In some cases, ESs generated by informal unmanaged green spaces are even higher than those generated by the establishment of urban parks [86]. Despite the relatively vast body of scientific information about ES generation during brownfield recovery, less is known about the role that phytoremediation could play in this context. In fact, many differences may occur when greening a brownfield using a more engineering-oriented approach. Most phytoremediation approaches use few plant species to target specific soil contaminants, and although some attempts have been done to enhance plant diversity by co-cropping different species [87], it is unlikely that floristic diversity during phytoremediation would be higher than a vegetation cover naturally established [88], where the distinctive spatial–temporal dynamics of urban brownfields induce a relatively high species diversity [89]. Another difference is that unlike for most soft brownfield re-use approaches, where the site can be entered, phytoremediation sites are normally inaccessible and, thus, several ESs related to the use of urban green spaces may be attenuated. Despite the long-term research on phytoremediation, few specific studies have been dedicated to quantifying ESs related to this green technology [90], whereas most research has focused on afforestation/reforestation with non-specific phytoremediation approaches [91,92,93]. The main potential ESs related to brownfield remediation are reported in Figure 1. 

### 3.1. Regulating Ecosystem Services

The phytoremediation of urban brownfields could add important benefits in terms of regulating environmental parameters, including soil, temperature, hydrology, biodiversity, noise attenuation, and carbon sequestration. 

#### 3.1.1. Soil Quality

Using living plants for the reclamation of urban brownfield can have a profound impact on soil quality and regeneration. Urban brownfields are formed as a result of anthropogenic factors (pollution, compaction, and loss of fertility) and natural factors of soil formation [94]. Unlike most conventional remediation techniques, which are associated with high soil disturbance [95], plants involved in phytoremediation have been shown to reduce soil disturbance, thus enhancing the carbon storage in soil as organic matter [96]. Different soil properties have been shown to improve following plant establishment on polluted sites, including chemical [97], physical [98], and biological [99] characteristics. The use of arbuscular mycorrhizal fungi in phytoremediation has been found to increase stress tolerance [100] and the heavy metal bioavailability for the plants, supporting their establishment on harsh sites [101]. Enhanced soil physicochemical properties were observed when phytoremediation was assisted by soil amendments. For instance, compost has been shown to improve soil physicochemical properties, including the increment in soil organic matter and nutrient content, at a Cu-contaminated site phyto-managed with different herbaceous species [102]. As in the case of compost, adding biochar to contaminated soils has been shown to increase soil pH, water-holding capacity, and soil fertility; reduce the mobility of plant-available pollutants; and promote vegetation establishment [103]. The establishment of a new vegetation cover on a brownfield also can reduce soil erosion by wind and leaching of soil-contaminating elements to groundwater [104]. Plants can create a physical barrier that holds soil particles in place. This action can be aided by root exudates, which reduce, by precipitation, the mobility of specific contaminants in the environment [105]. The benefits of phytoremediation related to soil quality are somewhat counterbalanced by some risks. The most common threat is represented by the application of mobilizing agents, which solubilize toxic soil contaminants that, when not promptly taken up by the plants, can be spread in the environment [106]. Though these uncertainties need further research, most phytoremediation approaches represent a valuable tool to enhance the overall soil fertility, thereby strengthening its ecological resilience to further disturbances.

#### 3.1.2. Urban Temperature

The establishment of a plant cover in an urban unvegetated area may lead to a significant mitigation in urban temperature, in particular by attenuating the urban heat island (UHI) phenomenon, i.e., severe temperature increases of several degrees (sometimes over 10 °C) compared to the surrounding areas [107]. In most cases, this action is achieved through direct shading, enhanced evapotranspiration, and thermal and optical properties specific to plants [108]. This action shows positive effects also in energy savings following climatization in city buildings [109]. Additionally, a vegetation cover in winter (especially evergreen trees) can represent a valuable windbreak protecting buildings from cold winds, therefore reducing energy consumption for heating [110]. It is well established that the cooling effect of vegetation depends on a combination of multiple factors, including structural (leaf colour and canopy structure) and functional (ecophysiological adaptations) traits of plants [111], environmental factors, and the size of the vegetated area [112]. Arboreal and herbaceous vegetation contribute differently to temperature mitigation. Trees appear to be more efficient in reducing high diurnal temperatures through the shading effect of their crown, whereas their canopy can retain heat at night by decreasing the movement of warm air and, thus, reducing emissions of long-wave radiation [111]. Another study has shown that woody vegetation (trees and shrubs) is able to reduce daily soil-surface temperatures in the summer by 5.7 °C compared to herbaceous vegetation and tends to maintain slightly higher temperatures in winter [113]. Other studies have pointed out that although both vegetation types are able to reduce the UHI in hot weather, grass showed a lower impact on local air temperatures and on human comfort, whereas trees provided effective and substantial local cooling [114]. Despite the vast body of literature on the thermal effects of urban vegetation, no reported information, however, is currently available on whether and to what extent phytoremediation can contribute to mitigating urban temperature, and therefore, any effect on temperature is not easy to predict. Most herbaceous hyperaccumulator species commonly used in phytoremediation are likely to respond as normal herbaceous vegetation. On the other hand, fast-growing woody species in short rotation are not very predictable. These stands, which are harvested over very short intervals (1–3 years), result in a very dense shrubby structure (up to 30,000 plants ha^−1^) and show very high evapotranspiration rates [115]. This will likely induce cooler air temperatures around the site, but the high density of the canopy structure could also decrease dissipation through soil irradiation at night. In addition, the high evapotranspiration rates of these species could be severely reduced by some sort of stress response to a high level of soil contamination in some spot of the brownfield [116]. Therefore, further research work is needed to clarify the effect of these specific plant stands on temperature regulation at the urban scale.

#### 3.1.3. Urban Hydrology

Flooding represents one of the main hazards in modern towns and cities. The increased number of flood events per year occurring in urban areas is mainly due to two factors: a higher frequency of extreme rainfall under current global climate change events [117] and human-induced alterations in land cover [118]. Soil sealing during urban sprawl is the main anthropogenic activity that reduces water infiltration and causes stronger surface run-off and flooding [119]. Green urban area expansion represents, among others, a valid technical solution to be implemented in order to attenuate this phenomenon [120]. The reduced risk of flooding displayed by vegetation is due to its ability to intercept, retain, and infiltrate rainwater. Plants capture and evaporate large amounts of rainwater directly via their surface tissues, and intercept and transfer large amounts of water from the soil to the atmosphere via transpiration [121]. In addition, due to low surface-soil bulk density, vegetation contributes to the infiltration of stormwater, resulting in a reduction of flood frequency and severity [122]. Although several herbaceous and woody species have been selected for their capacity to develop large root systems appropriate for flooded sites, herbaceous vegetation is in general more suited to mitigating stormwater runoff via soil infiltration, whereas trees are more efficient, due to canopy interception, in reducing the amount of water reaching the ground [114]. In fact, though the degree of attenuation of stormwater runoff depends on different characteristics, including species, canopy density, plant size, bark structure, canopy storage capacity, planting density, and the presence/absence of foliage [123], the best-suited species to attenuate this risk are perennials showing high evapotranspiration rates and Leaf Area Index and elevated canopy density. It is noteworthy that most perennial woody species used for brownfield reclamation not only share an annual stormwater canopy interception rate very close to most urban forest species [124], but also have very high evapotranspiration rates, both when grown as a single tree and under high-density, short-rotation, coppiced plantations [125]. Moreover, the positive hydrological effects of fast-growing tree species are also linked to the reduced soil bulk density measured over the long term [98].

#### 3.1.4. Carbon Dioxide Sequestration

Although plants represent one of the major environmental carbon sinks, the direct contribution of urban vegetation to carbon sequestration has been found to be smaller compared to the anthropogenic emissions of cities [126]. In fact, only young, fast-growing urban trees display a positive net carbon dioxide sequestration (CO_2_), which, unfortunately, decreases as the plants mature [127]. In addition, the maintenance of urban trees (e.g., pruning, fertilization, irrigation, and removal of dead leaves) also creates CO_2_ emissions [128]. However, urban vegetation, especially trees, can actively contribute to carbon sequestration via soil organic matter accumulation [129] and can contribute to reductions in CO_2_ emissions from fossil fuel combustion by decreasing the cooling and heating demand of city buildings [130]. Hence, the actual direct benefit of urban vegetation on carbon sequestration is still under active investigation [131]. From this perspective, an estimation of the contribution that phytoremediation stands can provide to a carbon budget is very difficult. While it is universally established that under environmental stress conditions, the photosynthetic activity of plants is negatively affected, thus reducing the net carbon assimilation [132], the extent of environmental stress conditions in most brownfield sites is not easy to predict. Evidence suggests that contamination in urban brownfield sites is rather low, and as such, the detrimental physiological effects are attenuated on most woody species currently used for phytoremediation [133]. In addition, tolerance to some type of contamination (e.g., heavy metals) can also be enhanced by mycorrhizal associations at the root level, which frequently occur with phytoremediation tree species [134]. Overall, it seems realistic that some phytoremediation species used for brownfield reclamation will contribute to carbon sequestration. Because these species are frequently managed in short-rotation coppice, thus kept under a juvenile status, they exhibit fast growth and high carbon sequestration rates [135]. On a heavy-metal-polluted brownfield, willow was found to sequester higher amounts of CO_2_ than maize and rapeseed [68]. Poplar and willow grown on a heavy metal phytoextraction site were estimated to stock up to 26 Mg ha^−1^ CO_2_ in woody biomass [136]. Other potential benefits are associated with the buildup of organic matter in the soil. Recent studies have shown urban brownfields to be capable of removing 4–59 Mg CO_2_ ha^−1^ yr^−1^ through direct precipitation of inorganic carbon [137]. Moreover, the adding of waste compost to soil under different species during brownfield reclamation has been proven to further enhance the build-up of long-term soil organic matter [138]. Another opportunity for CO_2_ mitigation is the use of biomass feedstock from phytoremediation. Once a detailed ecotoxicological risk assessment is performed, the biomass obtained from these stands, especially where woody plants are used, could contribute to decreasing CO_2_ emissions by replacing fossil fuels and, thus, play an important role as environmentally renewable global energy suppliers [139].

#### 3.1.5. Biodiversity

The urban landscape is ecologically characterized by habitat fragmentation and is often associated with lower biological richness than natural ecosystems [140]. When plant species richness is high, it is often due to the occurrence of exotic plants that have been introduced accidentally or deliberately for ornamental purposes. Urban brownfields, which are primarily shaped by disturbance, show typical traits of early-stage secondary succession, in which communities are characterized by non-specialist opportunistic species. These communities are represented by annual plants that predominate the early developmental stages of a brownfield, followed by perennials that usually gain dominance in the succeeding stages [89]. Sometimes, resource limitation, which often occurs in urban brownfields (e.g., water, nutrient, and poor soil quality), can prolong the pioneering stages of the succession process [141]. However, the highly diversified ecological conditions can create a unique and impressive diversity of plant [142] and animal species [143], although the stress associated with high pollution levels recorded in some sites tends to favour the establishment of a few species that exhibit resistance traits [144]. A recent study on a brownfield in Spain abandoned for more than 20 years and polluted with arsenic and lead revealed the presence of a diverse tolerant flora, which included six plant species (i.e., *Lotus hispidus*, *Medicago lupulina*, *Plantago lanceolata*, *Dysphania botrys*, *Trifolium repens*, and *Lotus corniculatus*) [145]. Moreover, animal diversity is often affected by the renaturation of urban brownfields. A recent long-term study has shown that the unique ecological communities that can develop on abandoned brownfield allow for a high biological landscape diversity in terms of birds and insects [146]. From this perspective, phytoremediation represents an interesting tool to maintain high levels of biodiversity during reclamation. Unlike most traditional remediation technologies, which are associated with high environmental footprints, including a loss in biodiversity, phytoremediation has less of an impact [147]. While the establishment of a phytoremediation cover on brownfields requires appropriate land preparation that may have negative effects on some soil animal species, thereby leading to an initial decrease of biodiversity [148], over the long-term, this loss of biodiversity is generally only temporary. For example, stands of Salicaceae frequently used for phytoremediation have been shown to display higher plant species richness than agricultural land [149], and sometimes even higher than grasslands and marginal grassland strips [150]. In this case, the use of dense tree or shrub stands on brownfields can also prevent the establishment of invasive plants [151]. Moreover, these types of vegetation cover show overall increases in the abundance of birds and mammals [152], butterflies [153], arthropods [154], and earthworms [155] than agricultural land and residual habitat (i.e., urban areas). Furthermore, choosing different genotypes with varying growth habits can be helpful in creating different habitats, thereby attracting a larger diversity of animals [156]. Although some agronomic operations, such as chemical weed control, which suppresses understory vegetation, may temporarily reduce animal diversity by simplifying the heterogeneity of the habitat [157], most phytoremediation techniques play an important role in enhancing microbial diversity in the soil and increasing the relative abundance of plant-growth-promoting bacteria [158]. Interestingly, some authors have reported that the fungal community composition was directly related to the willow phylogeny following a phytoremediation study using various willow species on the site of a former petrochemical plant [159]. Enrichment in bacterial community structure and diversity was also observed where phytoremediation was supported by the incorporation of inorganic/organic amendments [160].

#### 3.1.6. Air Pollution

Many cities worldwide are currently experiencing severe air pollution as the most serious hazard for human health. Most of the urban air pollutants, which includes particulate matter (PM_10_, PM_2.5_, and PM_<1_), NO_x_, SO_x_, carbon monoxide, and ozone, originate from car traffic and transportation [161]. Air quality in urban areas is strongly affected by the presence of vegetation and its structure, and it is now widely acknowledged that vegetation has positive effects on the air quality of urban areas, thereby improving the liveability levels [162]. In the urban context especially, vegetated areas intercept, modify, and reduce the fluxes of air pollutants through a filtering action, both via the deposition of solid pollutants on leaf surfaces and the uptake of gaseous pollutants by stomata. While surface deposition is the quantitatively predominant mechanism for the attenuation of solid air pollutants, gaseous pollutants, such as O_3_, SO_x_, and NO_x_, are most likely removed via leaf stomatal uptake [163]. The ability of urban vegetation to intercept air pollutants depends on many factors, including physical urban traits (e.g., shape and size of streets), and traits related to vegetation, such as leaf longevity and phenology [164], leaf size and shape [165], and foliage density and porosity [166]. Moreover, leaf functional traits (including leaf surface free energy, single leaf area, surface roughness, specific leaf area, epicuticular wax content, and width-to-length ratio) are among the most important in determining the actual air pollution interception by vegetation in urban areas [167]. Although it is likely that the establishment of vegetation on a brownfield site could show some effect on intercepting airborne particles by acting as physical barriers, evidence is still lacking. Some woody species (e.g., willow, poplar, eucalyptus, etc.) used for environmental reclamation purpose are characterized by a shrubby structure and show similar traits (e.g., high canopy densities, leaf area index, etc.) than many vegetated barriers commonly used for air pollution attenuation along roads and highways [168]. For instance, evergreen shrub species, such as *Osmanthus* spp., *Nerium* spp., *Eucalyptus* spp., and *Mimosa* spp., are likely to show higher efficiency in airborne particle filtration than willows and poplars due to their ability to intercept and retain air pollutants all year round [169]. By contrast, plants can sometimes contribute to air pollution. They can enhance the air PM concentration through the pollen released at specific times of the year [170]. The negative effects of pollen on human health will be discussed in a subsequent section (see Section 3.3.3). Plant leaves may release numerous biogenic volatile organic compounds (BVOCs) that react with atmospheric NO*_x_* and contribute to the formation of O_3_. In addition, BVOCs can contribute to PM_2.5_ formation, thereby reducing the overall air quality in towns [171]. Unfortunately, some common species used for brownfield reclamation (e.g., poplar) belong to the high-BVOC emitters and can negatively affect air quality if planted in very large numbers [172]. The negative impact on air quality could be also exacerbated by the fact that significant amounts of BVOCs can be emitted in response to environmental stresses [173], including soil pollution. Therefore, phytoremediation approaches that make use of these plants on heavily contaminated soils could represent a potential source of atmospheric pollutants. However, most urban brownfield areas are usually not so contaminated as to trigger a severe stress response in plants [174], thereby reducing BVOC emissions and their negative impact on the air quality. Future investigation is required into this specific topic.

#### 3.1.7. Noise Attenuation

Urban greenspaces show noise-absorption properties and can reduce road traffic noise to a higher degree in comparison to most artificial barriers [175]. These properties are determined by the coexistence of physical [176] and psychological [177] factors. The presence of both soil and plants represents the main physical factor affecting noise attenuation. Vegetation consists of a multilayer structure, containing high amounts of both living and decayed materials (leaves, needles, branches, and decayed trunks). It is well established that the soil under vegetation covers has a pronounced influence on reducing low-frequency sound propagation [178]. Further, vegetation shows the potential of noise reduction, which depends on the species and structure of the stand. Noise attenuation by woody vegetation along streets has been positively correlated with the height and depth of the stand [179], and it also depends on plant density [180]. To date, noise attenuation in phytoremediation stands along streets and highways has never been assessed. Only a few published studies have reported noise attenuation for the most common woody species used for phytoremediation. Black poplar (*Populus nigra* L.) fences have been tested for noise attenuation along a highway in Erzurum, Turkey, showing positive results [181]. Less promising performances have been provided by *Eucalyptus camaldulensis* (Dehnh, 1832) fences near Alexandria (Egypt) [182]. Despite the lack of information for specific phytoremediation crops, it is not difficult to predict higher noise attenuation performances for approaches that make use of high-density shrubby tree species compared to herbaceous plants.

### 3.2. Provisioning Ecosystem Services

Polluted brownfields under reclamation using green approaches could potentially provide different provisioning services related to the plant species that are used. While using plants grown on a polluted site for food/fodder production is probably unrealistic [183] for safety reasons, much more realistic is their use for energy production within the emerging circular economy framework [184].

#### 3.2.1. Biomass for Bioenergy

One of the most promising provisional services related to the phytoremediation of brownfields is the possibility to use the lignocellulosic biomass issued from the site for energy purposes. The use of brownfields for biomass production, instead of areas where food crops are produced, could address the issue of the food versus fuel debate [185]. The potential of fast-growing woody plant species as sources of biomass, with high yield potential, low conversion and production costs, and an energy-efficient and sustainable value chain, has been well established [186]. Many species used in brownfield phytoremediation, such as poplars and willows, can produce high amounts of biomass, even on polluted sites [187]. The lignocellulosic biomass issued from these sites can potentially be used either as woodchips to provide energy for heat and electricity production or converted into a biofuel (bioethanol) using a variety of methods that differ largely in the way cellulose is hydrolysed [64]. Although purification equipment is usually effective in reducing the environmental risks, some concerns have been raised about the danger of spreading some toxicants (i.e., heavy metals) into the environment via fly ashes during the combustion process [188]. Previous studies have demonstrated that the concentration of some contaminants (heavy metals) in phytoremediation-borne biomass could be significantly higher than those of a reference biomass [78]. Consequently, it is crucial to better understand the fate of potential pollutants in the currently used fractionation processes and the possible dispersion of hazardous contaminants in the environment during the treatment.

#### 3.2.2. Bioindustry

Unlike the production of thermal energy, the conversion of contaminated biomass into added-value compounds and materials provides a new idea for the green treatment of contaminated biomass and is beneficial to the improvement of phytoremediation technology with fewer environmental and health risks [189]. Many species used in PE naturally produce several compounds suitable for industrial uses. For instance, willows (genus *Salix*), with 330–500 species worldwide, are a valuable source of biologically active compounds, such as flavonoids, phenolic and non-phenolic glycosides, organic acids, sterols, terpenes, and lignans, all with high economic potential [190]. Since plants growing on TE-contaminated hotspots are subjected to multiple environmental stresses, and phytochemical production in plants is enhanced by them, PE hotspots may become active, added-value phytochemical factories that enhance the overall environmental and economical values of PE. Phytochemicals are a growing revenue-generating industry. Plants can produce over 8000 phenolic compounds to perform a wide range of functions, including abiotic and biotic stress tolerance [191], many of which have commercial uses. For example, condensed tannins can be used as green alternatives to synthetic compounds used in adhesive production [192], as well as environmental-friendly bioflocculants and biocoagulants [193]. Lignans are thought to be effective in mammals in vivo as antioxidants, having potential for cancer chemoprevention, as well as anti-inflammatory activity [194]. They are extractable from biomass without negatively impacting other end uses. Many of the plants used for PE have the potential to produce a large array of phytochemicals. Even some herbaceous species, such as lemongrass, can provide heavy-metal-free, value-added chemicals (e.g., essential oils) after being processed by steam distillation [195]. The prospect of integrating value-added renewable chemicals as a supplementary component of the crop’s value has been shown to be feasible under non-stressed conditions [196]. However, evidence is still missing on whether and to what extent plants grown in a heavy-metal-polluted environment would enhance their phenolic compound yield, thus adding economic value to a site under PE.

### 3.3. Cultural Ecosystem Services

#### 3.3.1. Aesthetics

The aesthetic function can be easily associated with any approaches aimed at increasing the green vegetated area in the urban context. In this sense, all urban vegetation used to create pleasing visual compositions and to provide perfume and auditory effects shows an aesthetic value [197]. Urban vegetation, especially trees and shrubs, is used extensively throughout ornamental horticulture and is particularly appreciated because of its inherent beauty based on the structure, form, foliage pattern, and changing nature of the fruit, flowers, and leaves [198]. Stem height, canopy size, leaf colour, branching height, and canopy density have been found to be the most important traits in predicting the public’s aesthetic preference for trees in urban contexts [199]. Trees and shrubs have been shown to enhance most people’s aesthetic experience in the short and long term compared to flowers and, more generally, herbaceous vegetation [200]. As such, green technologies, including phytoremediation, unlike most conventional engineered techniques, have the potential to be aesthetically appealing for citizens, thereby increasing the overall value of the approach. Moreover, some plant species currently used for phytoremediation show some interesting traits of particularly high aesthetic value. For instance, sunflower, which is currently used as a phytoextraction annual plant [201], is also popular as an ornamental plant species for its highly aesthetic features [202]. Likewise, some species of willow used in phytotechnologies display a remarkable range of bark colour, from dark brown and purple to light yellow, providing a visually pleasing scene in winter, especially when planted in clusters [203]. In other common phytoremediation species, such as eucalyptus, the colour and shape of the bark vary greatly, not only among species, but with age [204]. Among these species are those with the capacity to be managed as short-rotation coppice, and as such, can be established at high densities along roads or on the edges of reclamation sites, creating a living visual barrier to screen unsightly objects and enhance the overall aspect of the area.

#### 3.3.2. Social Cohesion

Likewise, any approach aimed at brownfield reclamation phytoremediation can contribute to enhancing community cohesion. However, the use of green technologies could be even more successful if properly managed upon implementation. Evidence exists that the stakeholder engagement during planning and management is critical when proposing phytoremediation because this green approach is suited for sites where multiple end-uses are often envisaged [205]. Social cohesion could be attained through different strategies. Citizen science, where stakeholders from the non-scientific community are invited to participate in a research project in both scientific thinking, management, and data collection, is a very useful tool [206]. The citizen science approach has been used to address environmental pollution concerns through the collection of data for environmental management. This approach has been successfully used to improve the public’s understanding of air pollution and eventually reducing their personal exposure to contamination [207]. Brownfield reclamation through phytoremediation offers a huge potential for citizen scientist programs. First, phytoremediation requires a long timeframe to operate, frequent sampling campaigns to collect data, and a level of financial support that is, in many cases, limited [208]. The involvement of the non-scientist public in all aspects of a phytoremediation project (planning, implementation, maintenance, and evaluation) could result in increased public awareness about contaminated sites and green sustainable solutions to address environmental hazards and emphasize the important social role of learning about the remediation/reclamation of soil contamination. Stakeholders, if properly trained, can also be engaged in the management of some operations, such as stand establishment (e.g., tree planting) and/or maintenance (e.g., watering and weeding), which stimulates cooperative working, mutual learning, and experience-sharing, thereby increasing the overall social acceptability of a reclamation project [209]. Despite these positive features, some aspects of the green remediation of urban brownfields are still unclear. Some research has shown that the clean-up and revalorization of urban brownfields may increase the risk of gentrification, whereby lower-income communities are displaced elsewhere due to increasing the overall cost-of-living [210]. This phenomenon should be offset, and in this regard, new governance modes and larger-scale participation might be a step in the right direction to overcome this challenge. However, the political and power aspect that is inherent within inequality issues needs to be simultaneously addressed, as demanded by some researchers [211].

#### 3.3.3. Effects on Health

There is increasing evidence that green spaces in urban areas produce measurable benefits on psychological and physical health, including daily stress attenuation [212], increased self-discipline [213], and decreased anxiety, stress, and depression [214], and a generalised improvement of health conditions [215]. Although these functions are mostly associated with the full physical fruition of city green spaces (for example, walking, resting, and running activities within the green space), there is also increasing consensus that specific benefits related to human health can be provided by urban greening indirectly, without having to physically visit these spaces. For instance, during the recent COVID-19 pandemic period, the beneficial effect of a green view on people’s mental health has been proven to be stronger than that of the direct use of greenspace [216]. Other studies have shown that the green view through windows is associated with faster recovery from illness [217]. In this regard, green approaches for brownfield reclamation could provide these type of ecosystem services. In this sense, since the higher psychological benefits are reached when plant species richness is high, a mixture of different species/clones at the reclamation site would be strongly advisable. However, plants used for the reclamation of polluted sites should also be evaluated for any potential drawback they may show. One of the most common problems with plants is represented by their allergenic hazard through their pollen. Some woody species used for phytoremediation show some allergenic potential. For example, birch and alder, which are sometimes used in phytoremediation, are considered very highly allergenic, and their use in urban areas should be avoided [218]. Eucalyptus is another plant that may pose some concerns in terms of pollen allergenicity [219]. Some Salicaceae, such as *Populus* and *Salix*, which produce high quantities of highly allergenic pollen, are also considered risky [220]. However, these two genera are dioecious plants, and the choice of female plant material can thus attenuate the risk of pollen emission and the related health hazard. Moreover, the common management practice of short-rotation coppice keeps the stems of the plants to a juvenile phase, where flowering and pollen production is very rare. Another perceived hazard associated to phytoremediation is the release of small quantities of toxicants into the environment, which could represent a risk to human health. Though in some cases a release of some pollutants has been reported, the quality of the surroundings was not significantly affected [51]. In this regard, the management of the stand is of paramount importance to reduce such risks. For example, when operating with the goal of the phytoextraction of heavy metals from the soil, all aerial parts of the plant should be harvested before leaf shedding. This helps also in removing pollutants from the system entirely and avoiding the return of the pollutants to the soil via litterfall.

## 4. Conclusions

The phytomanagement of polluted brownfields may represent an avenue to meet the current sustainable development and planning goals for modern urban areas. Nevertheless, its practical application on a full-scale is still challenging due to a number of technical constraints that must be better understood and eventually overcome. In the meanwhile, a new perspective for looking at this approach is proposed that aims at valuing all potential side ecological services associated with this phytotechnology. Furthermore, by taking the type and intensity of pollution within sites into consideration, a system of prioritization of the different ESs possible could be created to determine the specific phytotechnology applied. For instance, areas of low pollution presenting lower risk for human health could prioritize social and cultural ESs, such as providing open green spaces, opportunities for community engagement, and environmental education in an urban setting. In these cases, the specific needs of the local community should be accessed to see how the site could be best incorporated into the social landscape of the city. In contrast, areas of intense pollution should instead prioritize provisional and regulating ESs, limiting direct community involvement. However, the priorities for intensely polluted sites could change over time as soil conditions improve, and as several studies have shown, there are mental and physical health benefits to be derived from simple visual exposure to vegetated areas. These sites in particular should be accessed and planned with a long-term vision in mind, leaving room for shifting priorities from provisional to social and cultural. Although some phytomanagement techniques using woody species are likely to provide similar services to those of urban forests, such as the specificity of most brownfields (i.e., pollution, inaccessibility, and harsh environmental condition for plants), a better understanding of the extent of these services under these particular environmental conditions is fundamental and represents one of the research topics to be investigated by multidisciplinary teams in the near future. Given the long-term investment and timeframe of these projects, the actual related benefits (and sometimes hazards) associated with phytomanagement should be carefully considered. While ESs emanating from phytotechnologies provide both tangible and intangible products, the risks and costs associated with the management of polluted biomass and increased allergens should be evaluated, and solutions and alternatives found, aided by improved technologies for waste management and greater knowledge of plant responses to abiotic stresses. Future studies focused on evaluating the efficacy and efficiency of phytotechnologies should aim to incorporate an analysis of the associated ecosystems systems, and where possible, provide opportunities for long-term monitoring. This comprehensive approach evaluating the environmental, social, and economic costs and benefits of phytoremediation and continually building on a foundation of information from field-based trials could allow this green technology to be used on a larger scale in our cities for their sustainable, brownfield regeneration processes.

## Figures and Tables

**Table 1:** The most common phytoremediation approaches.

Mechanisms of Phytoremediation	Description	Contaminant Type Addressed	Plant Species	Reference
Phytoextraction	Plants uptake pollutants via their roots and accumulataltion in aerial biomass whose harvest allows progressive removal from the soil	Inorganic pollutants (As, Cd, Cr, Cu, Ni, Pb, Se, Zn)	Hyperaccumulators (*Noccaea caerulescens*, *Brassica juncea*, *Alyssum* spp., *Arabidopsis helleri*, *Pteris vittata*, *Sedum plumbizincicola*, *Arabidopsis thaliana*) Fast-growing trees (*Populus* spp., *Salix* spp. *Eucalyptus* spp.)	[30,31,32,33,34,35,36]
Phytostabilization	Plants produce specific metabolites which reduce the solubility and mobility of contaminants within the rhizosphere	Inorganic pollutants (Al, Co, Cu, Cr, Fe, Mn, Mo, Pb)	*Acanthus ilicifolius*, *Agrostis capillaris*, *Arundo donax*, *Atriplex halimus*, *Brassica juncea*, *Populus deltoides*, *Jatropha curcas*, *Lolium perenne*, *Miscanthus sinensis x giganteus*, *Pteridium aquilinum*, *Ricinus communis*, *Salix purpurea*	[37,38,39,40,41]
Phytodegradation	Plants, frequently assisted by microorganisms, take up and transform contaminants into less harmful compounds	Organic pollutants (petroleum hydrocarbons, polycyclic aromatic hydrocarbons, pesticides)	*Tagetes patula*, *Aster amellus*, *Portulaca grandiflora*, *Aster amellus*, *Iris dichotoma*, *Gaillardia aristata*, *Echinacea purpurea*, *Festuca arundinacea*, *Medicago sativa*, *Cytisus striatus*, *Nerium oleander*, *Ricinus communis*, *Populus* spp., *Salix* spp.	[42,43,44,45,46,47,48]
Phytovolatilization	Plants take up the pollutant and transpire it to the atmosphere as a gas, hence removing it from the site	Inorganic pollutants (As, Hg, Se) Organic pollutants (trichloroethylene, tetrachloroethylene, MTBE)	*Pteris vittata*, *Arundo donax*, *Dittrichia viscosa*, *Oryza sativa*, *Zea mays*, *Brassica juncea*	[49,50,51,52,53]
Rhizodegradation	Soil contaminants are broken down by external plant processes mediated by microbial activity	Organic pollutants	*Vigna unguiculata*, *Helianthus annuus*, *Zea mays*, *Sorghum sudanense*	[54,55,56,57,58,59]

## Data Availability

No data are available for this research.
